# Can Clear Aligners Release Microplastics That Impact the Patient’s Overall Health? A Systematic Review

**DOI:** 10.3390/ma18112564

**Published:** 2025-05-30

**Authors:** Adriana Assunta De Stefano, Martina Horodynski, Gabriella Galluccio

**Affiliations:** Department of Oral and Maxillofacial Sciences, School of Dentistry, “Sapienza” University of Rome, Via Caserta, 600161 Rome, Italy; adriana.destefano@uniroma1.it (A.A.D.S.); gabriella.galluccio@uniroma1.it (G.G.)

**Keywords:** microplastics, dental materials, clear aligner appliances, Invisalign, health, systematic review

## Abstract

This systematic review aims to further current knowledge on the effects of microplastics from orthodontic clear aligners, identifying potential implications for human health and providing a basis for further research and development of alternative materials. A literature search to find all peer-reviewed citations relevant to the review topic was conducted in the following databases: PubMed, Scopus, Web of Science, and Cochrane Library on 31 December 2024. A manual search of grey literature was also performed. There were 62 citations retrieved by the search query, and 11 were selected for inclusion in the review. Four selected studies were in vitro, while seven were in vitro following intraoral material aging studies. Ten studies evaluated the surface morphology of the material after aging, among the mechanical characteristics assessed, while only one article evaluated the chemical characteristics and size of the microplastic particles released from the aligners after simulated in vitro use. Discussion: From the evaluation of the studies included in this review, it is possible to state that there is a gradual increase over time in the surface roughness of the material, and modifications occurred in the morphology and surface topography of the aligners. Furthermore, it emerged that dispersion of microplastics occurs during the use of different types of aligners, with microplastic particle sizes ranging from 5 to 20 μm The findings suggest that clear aligners may cause microplastic dispersion in saliva during therapy, and this could cause a problem for the general health of patients, due to the absorption or ingestion of these released molecules. Further research is needed to fully understand the extent of microplastics released from aligners and to find alternative materials that can reduce this occurrence.

## 1. Introduction

The treatment of dental malocclusions has been revolutionized, especially with the introduction of clear aligner therapy, which offers an aesthetic and comfortable alternative to traditional metal braces [1,2].

The clear aligner therapy usually consists of a series of tight-fitting clear plastic trays that cover the dentition [3]. The patient must always wear these trays except when eating and brushing teeth, and the protocol of use is 22 h a day, from 7 or 14 consecutive days for each aligner [4].

The composition of the material used to manufacture a clear aligner (CA) is determined by the manufacturing process, which can be divided into two types: the traditional vacuum thermoforming method with thermoplastic molding on physical models, and direct 3D printing without the use of intermediate physical models [5].

Although there are numerous CA systems on the market today, the thermoplastic molding method is still the most widely used to produce commercial clear aligners and in-house production aligners [6]. Thermoformed aligners represent a well-established and reliable solution with relatively low production costs, although they offer lower precision and involve a more complex manufacturing process. In contrast, 3D-printed aligners provide higher precision, advanced customization options, and more efficient production, though they come with higher initial costs and require certified biocompatible materials [6].

Thermoplastic polymers are frequently used in the production of clear aligners. Polyethylene terephthalate (PET) and its non-crystallizing amorphous copolymer, polyethylene terephthalate glycol (PETG), are widely used in the commercial production of clear aligners because of their excellent mechanical and optical properties [1,6,7]. Thermoplastic polyurethane (TPU), a highly versatile polymer composed mainly of di- and tri-isocyanates and polyols, is another extremely versatile polymer that has many beneficial properties, such as superior mechanical and elastomeric qualities, chemical and abrasion resistance, adhesion properties, and ease of processing [1,6,7].

Tera Harz TC-85 (Graphy, Seoul, Republic of Korea), a photopolymerizable polyester-urethane polymer, is currently the only commercially available 3D-printable material that satisfies the requirements for the direct 3D printing technique [6].

Thermoplastic polymers used as CA material are biocompatible and must be free of substances that could cause harmful local or systemic reactions [8,9]. However, long-term use of these devices in the oral cavity raises questions regarding their safety and potential risks to health [10]. Clear aligner materials are subject to aging and can be stressed by cyclic masticatory or parafunctional stresses, changes in salivary flow and composition, changes in the oral microbiota, and changes in the temperature of the oral cavity; these conditions affect their mechanical and physical properties [11]. The thermoplastic material may degrade chemically and physically when exposed to the oral environment for a long time [12]. In general, aging reduces fit accuracy, transparency, and biomechanical performance, which can compromise orthodontic outcomes if not managed appropriately [11].

Considering that patients wear CAs for significantly prolonged periods of time, this degradation could promote the release of microplastic particles, potentially affecting oral health, or, once ingested or inhaled, they could accumulate in body tissues, potentially exposing patients to long-term adverse effects on overall health [13].

Microplastics are any synthetic solid particle or polymeric matrix, with regular or irregular shape and with size ranging from 1 μm to 5 mm, of either primary or secondary manufacturing origin, and which are insoluble in water [14].

Microplastics have been linked to several human health risks [15]. Recent studies indicate that these particles may be ingested or absorbed by oral tissues, potentially leading to a range of health risks and can inducing inflammatory responses and oxidative stress and possible DNA damage (genotoxicity) [16]. Some plastic additives or unreacted monomers may act as endocrine disruptors, interfering with hormonal balance, while long-term exposure could also impact the gut microbiota and immune system. Additionally, microplastics may carry adsorbed environmental toxins or heavy metals, contributing to bioaccumulation and increased toxic load in the body [15]. Such processes are linked to chronic diseases such as cardiovascular disease [17], respiratory disorders [18] and even some types of cancer [19]. In addition, the release of toxic chemicals from microplastics can exacerbate cellular damage by contributing to increased production of free radicals, unstable molecules that compromise cells in the human body, with potential impacts on cellular function and survival [20].

The effects of microplastics derived from orthodontic aligners are still being studied, and there are currently no specific guidelines to quantify safe exposure levels. However, evidence of inflammatory and toxic risks makes it urgent to better understand the effect of these particles on human health to inform both consumers and health professionals.

Based on these premises, the following research question for this systematic review was “Can clear aligners release microplastics that impact the patient’s overall health?” and the aim of the research was to deepen current knowledge on the release of microplastics from clear aligners, identifying potential implications for human health and providing a basis for further research and the development of alternative materials.

## 2. Materials and Methods

### 2.1. Protocol

The Preferred Reporting Items for Systematic Review and Meta-analysis (PRISMA-2020) criteria [21] were used in the formulation of this study. The protocol for this systematic review was registered on the INPLASY website (https://doi.org/10.37766/inplasy2025.5.0009) under the registration number INPLASY202550009, accessed on 5 May 2025.

### 2.2. Eligibility Criteria

Potentially relevant articles were selected based on these eligibility criteria: published in the period 2000–2024; written in the English language; abstract and full text available. The population, intervention, comparison, and outcome (PICO) approach was used to establish the inclusion criteria:

Population: All studies (in vivo or in vitro) investigating any thermoplastic CA material.

Intervention: Any type of orthodontic CA, any brand, material, and thickness, used or not used.

Comparison: A group of untreated patients used as a comparison group, or a control group compared to the experimental groups.

Outcome: Changes in the surface morphology of CA, release of microplastics, and adverse effects on the patient’s overall health.

Information sources, search strategy, and selection process:

A literature search to find all peer-reviewed papers relevant to the review topic was conducted in the following databases: PubMed, Scopus, Web of Science, and Cochrane Library on 31 December 2024. A manual search of grey literature was also performed.

The search strategy used for this systematic review was the keywords (“Clear aligner” OR “orthodontic aligner” OR “transparent aligners” OR “Invisalign”) AND (microplastic OR “micro plastic” OR nanoplastic OR nano plastic OR “surface morphology” OR “surface roughness”). The studies that were accessible in English were selected.

Based on the eligibility criteria applied, the search was performed by two researchers (D.A.A. and H.M.) who independently assessed the titles and abstracts of the retrieved articles. Subsequently, articles potentially satisfying the inclusion criteria for the review were retrieved in full text. Finally, a consensus was reached among the two reviewers (D.A.A. and H.M.) to include/exclude articles from the review.

### 2.3. Data Items and Collection Process

To identify the list of variables to be extracted, two researchers (D.A.A. and H.M.) collaborated to execute data extraction. The following data were extracted and organized in Microsoft Excel spreadsheets: title, authors, publication year, study design, sample size, observation period, outcome measures, assessment method, and results. The authors then conducted a consensus analysis of these findings so that they could be discussed in this review.

Research designs and outcomes were used to categorize the studies. Text and tables were used to analyze and report the data, providing an accurate narrative-style descriptive overview.

### 2.4. Risk of Bias Assessment

As clinical trials were not included in the review, a quality evaluation, such as CASP (Critical Appraisal Skills Program) or a similar tool, could not be conducted. The CONSORT (Consolidated Standards of Reporting Trials) checklist adapted to in vitro studies of dental materials by Faggion et al. [22] was used to assess the quality of the articles.

The authors of the present systematic review used this checklist for the evaluation of the quality of the in vitro studies; items from 5 to 9 were eliminated from the checklist due to the selected studies being in vitro, so there is a low risk of bias and no need for randomization, and the sample size is not so important (Table 1).

## 3. Results

### 3.1. Study Selection

**Figure 1:**
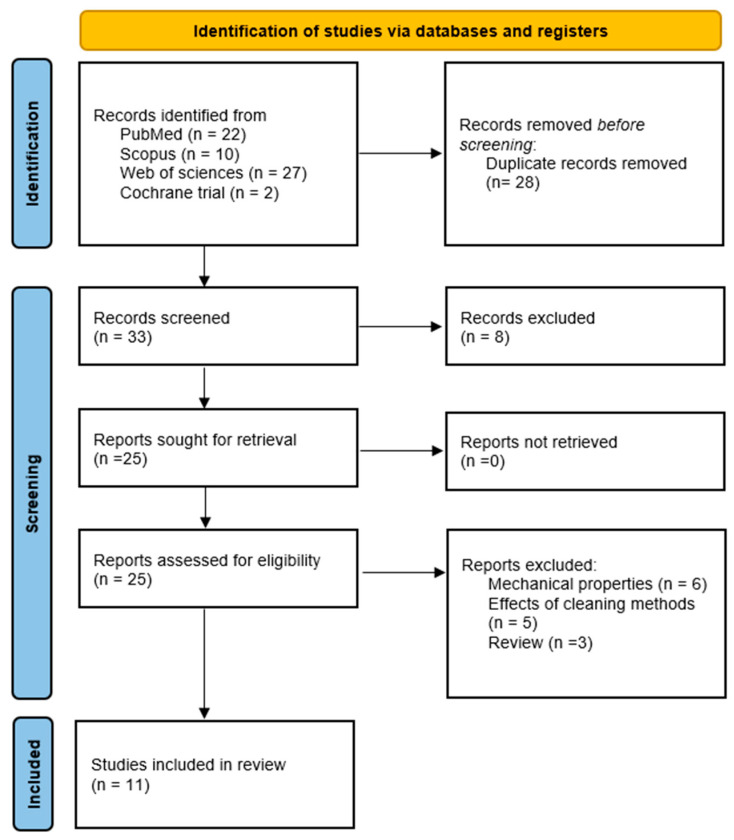
PRISMA flow diagram of the study selection procedure.

There were 62 citations retrieved by the search query. Following the removal of 28 duplicates and 8 articles that were clearly irrelevant based on their titles and abstracts, the complete texts of the 25 remaining articles were acquired. Fifteen were excluded for not being able to meet the inclusion criteria, and eleven were selected for inclusion in the review (Figure 1).

### 3.2. Study Characteristics

Four selected studies were in vitro [23,24,25,26], while seven were in vitro following intra-oral material aging studies [27,28,29,30,31,32,33]. The included studies were mostly published within the last five years: four in 2024, three in 2023, one in 2020, and one in 2019—the oldest article being from 2004—and were conducted in several countries. The descriptive characteristics of the included studies are outlined in Table 2.

Ten studies evaluated the surface morphology of the material after aging, among the mechanical characteristics assessed, while only one article evaluated the chemical characteristics and size of the microplastic particles released from the aligners after simulated in vitro use [26].

### 3.3. Risk of Bias in Studies

The results of the quality assessment are summarized in Table 3. According to the modified CONSORT checklist used, three articles obtained a score of 80%; six articles obtained a score of 90%, and the rest 100%. All 11 studies were included in the present review because they obtained a score of 80% or more.

### 3.4. Strategy of Data Synthesis

The narrative synthesis of data from the included studies will concentrate on two primary outcomes: (1) changes to the clear aligners’ surfaces that may indicate material degradation, and (2) the measurement of microplastic release during clinical usage. Methodologies, detection strategies, and published results will be compared using a structured thematic analysis with the goal of identifying recurring themes, methodological variations, and evidence gaps pertaining to the clinical behavior and environmental effects of aligners. Due to heterogeneity in study designs, detection methods, and outcome reporting, a meta-analysis was not feasible.

### 3.5. Results of Individual Studies

The analysis of individual results was divided according to the study objective into aligner surface roughness and the release of microplastics after use.

#### 3.5.1. Roughness Surface

##### In Vitro Study

Porojan et al. [25], Bhate et al. [23], and Mei et al. [24] performed in vitro studies to evaluate the variation of the surface of aligners in terms of surface roughness.

Poroijan et al. [25] used artificial saliva to simulate a 14-day aging process. The authors compared four types of PETG clear thermoplastic materials (Leone, Crystal, Erkodur, and Duran). The samples were submerged in artificial saliva with three different pH values: neutral (6.7), basic (8.3), and acid (4.3). The surface roughness was determined using a contact profilometer, and nanoroughness measurements were generated by three-dimensional profiles using an atomic force microscope (AFM). In terms of roughness, their findings indicate that following immersion and desiccation, the surfaces tended to be smoother on a microscale and more irregular on a nanoscale.

Bhate et al. [23] used distilled water to simulate a 14-day aging process by comparing clear aligners of PET-G (Duran material) and of TPU (Zendura material). Three distinct time points were used to measure the samples of the two groups: T0 was before thermoforming, T1 was after thermoforming, and T2 was after thermoforming and aging. Surface roughness was tested using a surface profilometer. According to their findings, the process of simulated intraoral aging affected the surface roughness in the TPU aligner.

Mei et al. [24] evaluated the biomechanical behavior of TPU aligners (Invisalign) by simulating aging in distilled water for 21 days. The biomechanical properties (flexural strength, translucency, surface roughness, hardness, and tensile strength) of the clear aligners were assessed each day. The surface roughness was measured using a profilometer. After seven and ten days, respectively, the clear aligners’ flexural strength and hardness dramatically declined. Throughout the 21 days of artificial aging, there was minimal variation in surface roughness, translucency, and tensile strength.

##### In Vitro Study Following Intra-Oral Material Aging

Eslami et al. [27] and Koletsi et al. [30] investigated the effects of 7 days of intraoral use on surface roughness in directly printed aligners (DPA) of resin (photopolymerizable polyester–urethane polymer) (Graphy, Seoul, Republic of Korea) and TPU (Invisalign).

Eslami et al. [27] used confocal laser scanning microscopy to investigate the effects of a week of intraoral use on the surface roughness characteristics of DPA and commercially manufactured Invisalign aligners compared to their unused control counterparts. The surface roughness parameters were examined using optical profilometry by Koletsi et al. [30]. According to both studies, directly printed aligners have a higher surface roughness increase than Invisalign.

Schuster et al. [32], Gracco et al. [29], Papadopoulou et al. [31], Fang et al. [28], and Lira et al. [33] evaluated TPU (Invisalign) aligners after intraoral use for 14 days. To observe the change in surface morphology, Schuster et al. [32] and Lira et al. [33] used an optical microscope (OM); Gracco et al. [29] and Fang et al. [28] used a scanning electron microscopy; and in the study of Papadopoulou et al. [31], an optical profilometer was used. According to these studies, Invisalign aligners retrieved from patients after the intraoral use for 14 days showed surface morphological changes in comparison to brand-new ones. The surface morphology of the used aligners showed microcracks, distortions, abrasion, and grooves. This decline occurred within the first week of clinical use and is not time-dependent.

##### Microplastic Release

Quinzi et al. [26] evaluated the potential release of microplastics from different PET-G aligners (Alleo; FlexiLigner; Lineo; Arc Angel, and Ortobel Aligner) and in TPU aligner (F22 Aligner; Invisalign) after 7 days of simulated aging process in artificial saliva and mechanical friction. The artificial saliva was filtered after seven days, and the filters were studied using Raman Microspectroscopy (RMS) and Scanning Electron Microscopy (SEM), respectively, to chemically characterize the polymeric matrix and to determine the size and quantity of the microplastics found. The authors found that all aligners studied released microplastics with a size range of 5 to 20 μm, with the highest number of microplastics in Arc Angel and the lowest in Invisalign. Microparticles with a diameter of 5–20 μm were found in all tested aligners and represented the largest group, with a percentage higher than 50%, except for F22 and Lineo (36% and 46%, respectively). A percentage range of 30–50% was detected for microplastics > 20 μm in all type of aligners, while microplastics < 5 μm were detected only in Alleo (17%), F22 (18%), and Invisalign (14%).

## 4. Discussion

This systematic review aimed to investigate the propensity of polymers used to produce clear aligners to fragment into microplastics during clinical intraoral use. The literature in this regard shows that during intraoral use, aligners suffer variations in their surfaces in the form of roughness, abrasion, microfractures, and grooves that are compatible with the release of material into the oral cavity in the form of microplastics.

Given the increasing use of clear aligners in orthodontic therapies in patients of all ages, it is becoming very interesting to investigate whether the release of microplastic material from these medical devices could be a risk to patients’ health.

The different thermoplastic materials used in the manufacture of clear aligners, which are mainly polymers of the thermoplastic polyurethane (TPU) and polyethylene terephthalate glycol (PET-G) types, have been described in the literature. However, different aligner companies use proprietary materials in the manufacture of aligners, so their exact composition is unknown [5]. The choice of thermoplastic polymer type depends on the material characteristics and properties that may affect the clinical performance of clear aligners during orthodontic treatment [1,8].

The protocol for using the aligners involves a use of 22 h a day from 7 to 14 consecutive days for each aligner [6]. Using aligners during all these hours inevitably leads to frequent contact between the upper and lower aligners, which results in wear or abrasion of the surfaces [34].

The studies included in this systematic review analyzed the changes in the surfaces of aligners after wear at 7 and 14 days, made of PETG, TPU, and DPA. The results show that after clinical use, there are surface changes as early as seven days in all types of aligners from different commercial companies, although Invisalign remains the most studied. Factors that can influence the modification of the surfaces of the aligners, in addition to the time of use—such as the type of diet, the oral cavity hygiene habits, the method of cleaning the aligners, and the influence of bad habits or behaviors such as bruxism—were not analyzed. However, these results suggest that if the surface of the aligner changes, it may release microparticles of material into the oral cavity.

Only Quinzi et al. [26] evaluated in vitro the effective dispersion of secondary microplastics from aligners of different commercial houses, made of PETG and TPU, subjected for 7 days to artificial chemicals and mechanical friction. Their results show that there is a release of microplastics of different sizes in a range between 5 to 20 μm, with different distributions depending on the material. Since Quinzi et al. [26] conducted a single 7-day assessment, it is not possible to quantify the amount of microplastics released day by day, nor the rate of release after day 7. New studies are needed to make these assessments.

Although studies on microplastics in the human body are limited, it has been shown in the literature that microplastics, when ingested, can cause damage to the digestive system, and that these particles can undergo structural changes by the microbiota of the intestinal system, also demonstrating an interaction with the system [35]. Before reaching the epithelium of the intestine, which is considered the main site of nutrient absorption, orally ingested plastic particles could pass through the epithelium of the stomach [36].

The size of microplastics is a determining factor in microplastics crossing the intestinal barrier. The larger ones remain in the intestinal lumen and are excreted via stool [37], but can cause disorders of the intestinal flora [38]. Smaller microplastics, up to a maximum size of 5–10 mm, can cross the intestinal barrier by endocytosis or by diffusion [13]. Microplastics can remain deposited in intestinal cells and cause inflammation and stress [13,38]. Only much smaller particles, up to 1.5 mm, could be distributed systemically through the bloodstream and potentially accumulate in various organs [37]. The presence of microplastics has been reported in the liver and has been associated with liver toxicity, and in the human spleen, but it is currently unclear whether microplastics can cause splenic dysfunction [39]. Particles circulating in the bloodstream can accumulate in the large arteries in the form of atheromatous plaques that correlate with cardiovascular events [17]. Microplastics can be considered an emerging risk factor for cardiovascular accidents, stroke, and death [40]. In addition to human organs, microplastics have been found in other human biological samples such as saliva, sputum, stool, urine, and breast milk; the presence of microplastics in these samples is due to passageways, storage, or excretion [16,37].

Although studies have demonstrated that microplastics are common in breast milk, the discovery of microplastics in meconium and infants’ stool raises additional concerns because, since they have been identified in the placenta, it is thought that they may be able to pass through the placental barrier, enter the fetal bloodstream, eventually make their way to the fetal intestine, and then be expelled in meconium [16,37].

Another aspect is the type of polymer that is most frequently deposited in tissues and detected in biological samples. The characteristics of microplastics— shape, size, color, and type of polymer—have been picked up by Roslan et al. [16] in their systematic review. They suggest that polypropylene and polyethylene microplastics can accumulate in various human tissues, but are most abundant in the digestive tract, placenta, and lungs. PET microplastics have been detected in blood vessels, heart, liver, spleen, lung tissue, placenta, breast milk, meconium, and newborn feces, while TPU has been reported in pulmonary tissue, placenta, breast milk, meconium, newborn feces, and stool.

The microparticles released by transparent aligners processed in PETG and TPU, according to the study by Quinzi et al. [26], have a size between 3 and 95 μm, with a higher percentage between 5 and 20 μm. Particles of this size could, as described above, cross the intestinal barrier and deposit in various organs, with a potential risk to patients’ health.

Among the limitations of this systematic review is that the potential release of microplastics during clinical use of clear aligners is only supported by in vitro studies, which do not consider all the real-world conditions to which clear aligners are subjected during intraoral use. Another limitation is represented by the fact that most of the studies included in the review are based on the evaluation of aligners from a few commercial houses, particularly Invisalign, but nowadays, many commercial companies on the market produce aligners, and there are also homemade aligners with non-standardized procedures. It should also be considered that many commercial houses use proprietary material to produce aligners, so there is no absolute knowledge of the type of polymer used.

Considering the limitations reported and the quality of the studies included, this review has clinical implications regarding the possible correlation between exposure to aligner microplastics and the health risk to the patient wearing them. The results suggest that the use of aligners should be reduced in pregnant and breastfeeding women. The protocols of aligner use must be reviewed, in light of the results obtained, to reduce the time of use of each aligner, to limit as much as possible the exposure to microplastics.

Based on these findings, further research is needed on the actual release of plastic microparticles from clear aligners. Although new studies should be conducted to evaluate other possible factors that may affect surface modification and subsequent release of material during clinical use of aligners.

It would also be interesting to assess whether different usage protocols can vary the potential release of microparticles and, to the same extent, to study whether new manufacturing materials can reduce the potential risk of microplastic release in the oral cavity.

## 5. Conclusions

The findings suggest that clear aligners may cause microplastic dispersion in saliva during therapy, and this could cause a problem for the general health of patients, due to the absorption or ingestion of these released molecules. Further research is needed to fully understand the extent of microplastics released from aligners and to find alternative materials that can reduce this occurrence.

## Figures and Tables

**Table 1:** Modified consort checklist for in vitro studies comparing different dental implant impression techniques.

Section	Checklist Item
Abstract	Item 1. Structured summary of trial design, methods, results, and conclusions
Introduction	Item 2a. Scientific background and explanation of rationale
Methods	Item 2b. Specific objectives and/or hypotheses
	Item 3. The intervention for each group with sufficient detail to enable replication
	Item 4. Completely defined measures of outcome, including how and when they were assessed
	Item 5. Statistical methods used to compare groups for primary and secondary outcomes
Results	Item 6. For each primary and secondary outcome, results for each group, and the estimated size of the effect and its precision
Discussion	Item 7. Trial limitations, addressing sources of potential bias, imprecision and, if relevant, multiplicity of analyses
Other information	Item 8. Sources of funding and other support
	Item 9. Where the full trial protocol can be accessed, if available

**Table 2:** Descriptive characteristics of the included studies.

Author (Year)	Study Design	Sample	Observation Period	Outcomes	Assessment Method	Results
Porojan et al. (2024) [25]	In vitro	Ten pieces of four types of PETG clear thermoplastic materials: Leone (L), Crystal (C), Erkodur (E), and Duran (D)were stored in artificial saliva with 3 different pH values: Group 1: neutral pH = 6.7. Group 2: basic pH = 8.3. Group 3: acidic pH = 4.3.	T0: “as-received aligner” T1: 14 days after simulated aging process.	Surface topographies: Surface roughness Surface nanoroughness	The surface topographies were analyzed on two length scales: The surface roughness was determined using a contact profilometer, and nanoroughness measurements were generated by three-dimensional profiles using an atomic force microscope (AFM).	On the microscale, the surfaces tended to be smoother after the saliva immersions, and on the nanoscale, they became more irregular.
Bhate et al. (2024) [23]	In vitro	Two groups with 12 samples per group: Group 1: polyethylene terephthalate glycol (PET-G). Group 2: zendura-polyurethane (PU).	T0—prethermoformed T1—after thermoforming T2—after thermoforming and aging by thermocycling (a total of 200 cycles over the course of 14 days)	Surface roughness Flexural modulus	The samples were immersed in distilled water at 37 °C for 24 h. Subsequently, thermocycling was performed. Surface roughness was determined using a surface profilometer. Flexural modulus was determined using a three-point bending test.	The aging process affected the surface roughness in Zendura (PU). The thermoforming and aging process resulted in reduced flexural strength in both Zendura (PU) and Duran groups (PET-G).
Mei et al. (2024) [24]	In vitro	A total of 63 new clear aligners (Invisalign, Align Co., Tempe, AZ, USA)	T0: “as-received aligner” T1: 21 days of simulate aging process in distilled water at 37 °C.	Surface roughness	Surface roughness was measured using a profilometer every day.	Surface roughness varied little during the 21 days of artificial aging.
Eslami et al. (2024) [27]	In vitro study following intra-oral material aging.	Four groups with 34 samples per group: Group 1: directly printed aligners retrieved after 1 week of intraoral service. Group 2: Invisalign aligners after 1 week of intraoral use. Group 3: unused directly printed aligners. Group 4: unused control Invisalign aligners.	After 1 week of intraoral usage	Surface roughness Surface porosity	Surface roughness and porosity measuring using confocal laser scanning microscopy.	An increase in the surface roughness and surface porosity of directly printed aligners following 1 week of intraoral usage.
Koletsi et al. (2023) [30]	In vitro study following intra-oral material aging.	Four Groups with 20 samples for the B and D groups and 12 samples for the A and C groups: Group A: “as-received” Invisalign aligners. Group B: clinically used Invisalign aligners (7 days). Group C: “as-received” 3D-printed aligners. Group D: clinically used 3D-printed aligners (7 days).	After 7 days of intraoral usage.	Surface roughness	Optical profilometry was employed to examine the following surface roughness parameters: amplitude parameters Sa, Sq, and Sz and functional parameters Sc and Sv.	Surface roughness differences existed between 3D-printed aligners and Invisalign in the retrieved status, as well as between the control and retrieved 3D-printed groups. Intra-oral exposure and function induced significant and substantial changes in surface roughness properties of “in-house”-fabricated aligners at all levels.
Schuster et al. (2004) [32]	In vitro study following intra-oral material aging.	Invisalign appliances were randomly selected from 10 patients before intraoral placement and after 2 weeks of intraoral usage.	T0: “as-received” aligner T1: After 14 days of intraoral usage.	Surface morphology	Bright-field optical reflection microscopy was used to study the surface morphology	The retrieved appliances demonstrated substantial morphological variation relative to the as-received specimens involving abrasion at the cusp tips.
Gracco et al. (2009) [29]	In vitro study following intra-oral material aging.	One “as-received” Invisalign^®^ aligner, One “as-received” Invisalign^®^ aligner immersed in artificial saliva for 14 days. Ten Invisalign aligners worn by 10 randomly selected patients for 14 days.	After 14 days of intraoral usage.	Surface morphology	Scanning electron microscopy and energy dispersive X-ray microanalysis were used to examine the surface morphology.	Aligners worn for 14 days had microcracks, abraded and delaminated areas.
Papadopoulou et al. (2019) [31]	In vitro study following intra-oral material aging.	Forty Invisalign^®^ appliances retrieved after the end of orthodontic treatment from different patients: Group 1: 20 aligners used for one week. Group 2: 20 aligners used for two weeks. Control group: 10 unused aligners.	After 7 and 14 days of intraoral usage.	Surface roughness	The Sa, Sq, Sz, Sc, and Sv roughness parameters of the internal surface of the aligner attachment area and the opposite lingual side were determined by optical profilometry.	The surface roughness of the retrieved groups (1 and 2) showed statistically significant differences compared with the control group, but without statistically significant differences between each other. The roughness variables of the as-received material were shown to be reduced after intraoral service demonstrating a wear effect. Aging has a detrimental effect on the surface roughness of Invisalign appliances, although this effect is restricted to the first week of clinical usage.
Fang et al. (2020) [28]	In vitro study following intra-oral material aging.	Two groups with twenty sample per group: Group 1: “as received” Invisalign aligners. Group 2: retrieved (2-week) Invisalign aligners.	After 2 weeks of intraoral usage.	Surface morphology	Facial one-third of maxillary central incisors from the aligners were cut vertically. Scanning electron microscopy and transmission electron microscopy were used to observe the changes in surface morphology.	The surface morphology showed some defects after the clinical use of 2 weeks.
Lira et al. (2023) [33]	In vitro study following intra-oral material aging.	Two Groups with 12 sample of Invisalign aligner for groups.Group 1: as received aligners. Group 2: retrieved Invisalign aligners used for 14 days.	After 14 days of intraoral usage.	Surface roughness morphology and topography.	The surface morphology was evaluated using an optical microscope (OM), scanning electron microscope (SEM).	The surface roughness of the material tended to increase, and modifications occurred in the morphology and surface topography of the aligners, characterized by the appearance of microcracks, grooves, and distortions.
Quinzi et al. (2023) [26]	In vitro	Two aligners from different manufacturers: Alleo (AL); FlexiLigner (FL); F22 Aligner (F22); Invisalign^®^ (INV); Lineo (LIN); Arc Angel (ARC), and Ortobel Aligner (OR).	After 7 days of simulated aging process.	Chemically identify the polymeric matrix Number and size of the detected microplastic	The aligners were immersed in artificial saliva for 7 days and stirred for 5 h/day, simulating the physiological mechanical friction of teeth. Then, the artificial saliva was filtered, and filters were analyzed by Raman Microspectroscopy (RMS) and Scanning Electron Microscopy (SEM), respectivel, y to chemically identify the polymericmatrix and to measure the number and size of the detected microplastic	RMS spectra revealed that AL, FL, LIN, ARC, and OR aligners were composed by polyethylene terephthalate, while F22 and INV ones by polyurethane. SEM analysis showed that the highest number of microplastics was found in ARC and the lowest in INV. Microparticles with a diameter of 5–20 μm were found in all tested aligners and represented the largest group, with a percentage higher than 50%. A percentage range of 30–50% was detected for microplastics > 20 μm in all the aligners, while microplastics < 5 μm were detected only in AL (17%), F22 (18%) and INV (14%).

**Table 3:** Results of the quality assessment of selected articles using the modified consort checklist for in vitro studies comparing different dental implant impression techniques.

Author	Abstract	Introduction	Methods	Results	Discussion	Other	Total
	1	2a 2b	3 4 5	6	7	8 9	
Bhate et al. (2024) [23]	yes	yes yes	yes yes yes	yes	yes	yes yes	10
Eslami et al. (2024) [27]	yes	yes yes	yes yes yes	yes	yes	yes no	9
Fang et al. (2020) [28]	yes	yes yes	yes yes yes	yes	yes	no no	8
Gracco et al. (2009) [29]	yes	yes yes	yes yes yes	yes	yes	//	8
Koletsi et al. (2023) [30]	yes	yes yes	yes yes yes	yes	yes	yes no	9
Lira et al. (2023) [33]	yes	yes yes	yes yes yes	yes	yes	no yes	9
Mei et al. (2024) [24]	yes	yes yes	yes yes yes	yes	yes	yes no	9
Papadopoulou et al. (2019) [31]	yes	yes yes	yes yes yes	yes	yes	yes no	9
Porojan et al. (2024) [25]	yes	yes yes	yes yes yes	yes	yes	no yes	9
Quinzi et al. (2023) [26]	yes	yes yes	yes yes yes	yes	yes	yes yes	10
Schuster et al. (2004) [32]	yes	yes yes	yes yes yes	yes	yes	//	8

## Data Availability

The datasets generated during and/or analyzed during the current study are available from the corresponding author on reasonable request.
